# From glass to pixel: Field restructuring, habitus crisis, and the revaluation of data in contemporary anatomic pathology—a sociological and autoethnographic perspective

**DOI:** 10.1016/j.acpath.2026.100302

**Published:** 2026-10-05

**Authors:** Hugo David Alfici

**Affiliations:** Service of Anatomic Pathology, Hospital Escuela de Agudos Dr. Ramón Madariaga, Posadas, Misiones, Argentina

**Keywords:** Autoethnography, Bourdieu, Digital pathology, Field theory, Habitus, Medical informatics, Pathology informatics, Structured reporting, SNOMED CT, Symbolic capital

## Abstract

The digitalization of anatomic pathology is a profound restructuring of the medical field — not merely a technological upgrade. Drawing on Pierre Bourdieu's theory of field and habitus, this work argues that nearly two centuries of methodological stability have produced a deeply sedimented professional habitus whose symbolic authority is now challenged by the emergence of technological, informational, and algorithmic capital. Resistance to digital transition is not technophobia. It is a structural response to the redistribution of historically accumulated professional capital — a phenomenon Bourdieu calls hysteresis. The traditionally unstructured narrative pathology report, long the primary vehicle of diagnostic authority, is simultaneously being displaced by structured data as a new form of capital. This process is not epistemologically neutral: controlled terminology systems such as Systematized Nomenclature of Medicine — Clinical Terms, natural language processing-based tools, and artificial intelligence algorithms construct rather than merely capture biological truth. Autoethnographic reflection from the author's dual practice — more than fifteen years as a pathologist and concurrent training as a medical informatics specialist — provides first-person evidence of this structural mismatch. The framework presented here is theoretical and autoethnographic in nature; it invites empirical follow-up. The future of the pathologist depends not solely on technological adaptation, but on the capacity to redefine symbolic capital at the convergence of morphology, structured data, and algorithmic interpretation.

## Introduction

The transition toward digital pathology has been examined primarily from technical and organizational perspectives: diagnostic validation, operational efficiency, interoperability, and artificial intelligence (AI) integration.^1^, ^2^, ^3^ The structural implications of this transformation for professional subjectivity and symbolic authority remain underexplored.

This work applies Pierre Bourdieu's theory of field and habitus^4^^,^^5^ to the digitalization of anatomic pathology. The shift from optical microscopy to digital environments constitutes a restructuring of capital distribution within the medical field. It generates tensions that reflect structural conflict — not mere resistance to technology.

The central hypothesis is that digitalization produces hysteresis: a structural mismatch between the historically constituted habitus of the pathologist and the new conditions of the field. This mismatch causes consequences for professional identity, diagnostic authority, and the value of the pathology report as a form of capital.

## Theoretical framework: Bourdieu's field theory applied to pathology

Pierre Bourdieu (1930–2002) was a French sociologist whose theory of social practice provides a rigorous framework for analyzing how professional expertise is acquired, valued, and threatened by structural change.^4^, ^5^, ^6^, ^7^ Three of his core concepts are central to this analysis.

*Habitus* refers to the durable, embodied dispositions that individuals acquire through sustained participation in a field. It is the professional “second nature” that experienced practitioners develop. The trained eye of the senior pathologist — integrating morphological patterns, clinical context, and diagnostic probability in a single glance, without conscious deliberation — is habitus in action. It is built slowly, through thousands of cases. It cannot be transferred by reading a textbook.

*Hysteresis* — borrowed by Bourdieu from physics, where it describes a system's delayed response to change — refers to the mismatch that arises when a field's structural conditions transform faster than its practitioners' habitus can adapt. In anatomic pathology, hysteresis manifests as friction experienced by senior pathologists whose morphological competence remains fully intact yet does not automatically translate into the new informational demands of digital workflows. Hysteresis-driven resistance is structural, not personal. It reflects a legitimate conflict over the redistribution of professional capital.

*Algorithmic capital* is a term introduced here to name an emerging professional resource: the competence to deploy, interpret, and critically audit AI-assisted image analysis tools, natural language processing (NLP)-based report mining systems, and predictive models. Like all capital in Bourdieu's framework,^8^ algorithmic capital is unequally distributed, confers competitive advantage, and is increasingly required for full participation in the contemporary pathology field.

*Autoethnography* is a qualitative research methodology in which the researcher's own lived experience serves as primary data.^9^^,^^10^ Rather than claiming the false neutrality of pure observation, autoethnography positions the researcher as simultaneously insider and analyst. In this work, that vantage point allows documentation of structural hysteresis from within the field itself.

**A methodological note** is warranted. This article is a theoretical and autoethnographic essay. It applies a sociological framework to a professional transformation and grounds its claims in the author's reflective practice — not in systematic empirical data. No survey, interview, or quantitative study is reported. The argument's strength lies in the coherence of its theoretical framing and the resonance of its autoethnographic evidence. Readers seeking empirical validation are encouraged to treat this work as a conceptual foundation for future studies. Table 1 summarizes all key Bourdieusian concepts used throughout this manuscript alongside plain-language equivalents and concrete applications to anatomic pathology practice.

**Table 1:** Bourdieusian concepts used in this manuscript and their plain-language equivalents for the pathology reader.

Bourdieusian term	Plain-language equivalent	Application to anatomic pathology
Field	The professional arena with its own rules and power structures	The professional space of anatomic pathology, with its own standards, hierarchies, and forms of recognized expertise
Habitus	Deep-seated professional instincts and ways of working	The trained eye, diagnostic intuition, and workflow habits built over years at the microscope; the pattern-recognition competence experienced pathologists embody
Hysteresis	When old habits no longer fit new conditions	The friction experienced by senior pathologists whose morphological skills do not automatically transfer to the structured data demands of digital workflows; resistance to change as a structural, not personal, phenomenon
Symbolic capital	Professional authority and prestige	The pathologist's recognized authority to issue definitive diagnoses; the trust clinicians place in the pathology report as the final word on tissue diagnosis
Incorporated cultural capital	Expertise that lives in the body, not on paper	The accumulated visual memory of thousands of histological cases; competence that cannot be obtained from textbooks alone but only through years of practice
Technological capital	Infrastructure and digital know-how	Whole-slide imaging scanners, LIS/DPMS platforms, cloud storage, and the institutional and personal capacity to deploy and use them effectively
Informational capital	Ability to structure and use data	Skill in producing structured pathology reports, applying synoptic templates (e.g., CAP checklists), and integrating molecular and clinical data into interoperable records
Algorithmic capital	Capacity to work with and validate AI tools	Competence in deploying, interpreting, and critically auditing AI-assisted image analysis, NLP-based report mining, and predictive models in pathology workflows
Autoethnography	Using one's own experience as research evidence	The author's reflective account of practicing as a pathologist while simultaneously training in medical informatics — providing first-person evidence of the structural mismatch described theoretically
Structural subversion	A historical shift that rewrites the rules of the profession	Virchow's cellular pathology (1858), which repositioned diagnostic authority from the bedside to the microscope; and today's digital turn, which similarly repositions authority toward data platforms and algorithms

AI: artificial intelligence; CAP: College of American Pathologists; DPMS, digital pathology management system; LIS: laboratory information system; NLP: natural language processing.

### Genesis of the field: from cellular pathology to scientific subdiscipline

The publication of *Cellular Pathology* in 1858 by Virchow^11^^,^^12^ marks the foundational moment of anatomic pathology as a scientific subdiscipline. The microscope became the privileged device of diagnostic evidence, repositioning the pathologist as the authorized agent for consecrating biological truth through morphological interpretation. In Bourdieusian terms, this constituted a structural subversion that reorganized the distribution of scientific and symbolic capital within the medical field.^6^^,^^7^

From that point forward, the pathologist occupied a unique epistemic position: the one who could see what others could not. That authority was grounded not in social status alone, but in a genuine technical capacity. It was earned, and then sedimented over generations.

### The visual habitus and technical stability

For almost two centuries, the optical microscope, paraffin-embedded histological sections, and hematoxylin and eosin staining have remained essentially unchanged.^13^ This extraordinary technical continuity has enabled the deep sedimentation of a professional habitus: a set of perceptual and cognitive dispositions oriented toward morphological interpretation under conditions of uncertainty.

Three structural characteristics define this habitus. First, the bodily incorporation of knowledge: visual training configures durable perceptual schemas. Second, the practical economy of scarcity: diagnosis is regularly produced with incomplete clinical information. Third, interpretive autonomy: wide margins of expert judgment operate in the production of diagnostic meaning. The habitus functions as a device of symbolic integration.

What the pathology literature calls the “pathologist's mindset” — the integrative, pattern-based, probabilistic reasoning that experienced practitioners apply at the microscope — maps closely onto what Bourdieu theorizes as habitus. The difference is analytical precision: habitus names not just the cognitive style, but its social origin, its embodied character, and its structural vulnerability when field conditions change.^4^^,^^5^

### Time as accumulated capital

The symbolic authority of the senior pathologist is closely linked to experience accumulated over thousands — often tens of thousands — of histological observations spanning years or decades of practice. This accumulated visual memory is not uniformly distributed across the profession. It is the distinguishing characteristic of experienced practitioners. Residents and early-career pathologists are still building this archive; it is the senior pathologist who most acutely experiences the structural tension of the digital transition, having invested most heavily in the form of capital now being contested.

The “trained eye” functions as a living archive: a form of incorporated cultural capital whose value derives from prolonged repetition of the act of looking. Methodological stability across two centuries has reinforced this intergenerational accumulation, producing a field with extraordinary internal coherence and symbolic legitimacy.^14^

### Digitalization and the transformation of the field

Digital pathology introduces an accelerated modification of the field's objective conditions. The glass slide becomes a replicable digital image, integrable in information systems and analyzable by algorithms.^1^, ^2^, ^3^ Three new forms of capital emerge: technological capital (digital infrastructure and computational capacity), informational capital (structured integration of clinical and molecular data), and algorithmic capital (predictive models and automated analysis).

The integration of diagnostic information, which historically occurred in the cognitive space of the pathologist, begins to externalize onto digital platforms. Diagnostic legitimacy no longer rests exclusively on incorporated cultural capital. It also depends on data processing capacity — a fundamental shift in the rules of the field.

### Habitus crisis, structural mismatch, and change management

The habitus is constituted in correspondence with relatively stable field structures.^4^^,^^5^ When these structures transform rapidly, hysteresis occurs: dispositions persist that no longer correspond to new field conditions. This generates a specific form of distress that is structural — not psychological.

Resistance to digitalization is not technophobia or nostalgia. It reflects a structural conflict over the redistribution of capitals and the redefinition of professional autonomy. The pathologist who resists is defending real, legitimate, functionally effective capital — capital that has not yet found its form of conversion into the new currency of the digital field.

From a change management perspective, the digital transition is frequently framed as an adoption problem: identifying barriers, designing training programs, and measuring implementation success.^8^ While valuable, this framing risks reducing a structural phenomenon to a logistical one. The hysteresis model suggests that effective change management in pathology must go beyond tool adoption. It must address capital reconversion: helping practitioners translate accumulated morphological expertise into new forms of valued competence. Resistance that is structural in origin requires structural responses — not merely training, but a redefinition of what counts as excellence. Figure 1 illustrates the zone of hysteresis across the preanalytical, analytical, and postanalytical phases of the anatomic pathology workflow, mapping where the traditional habitus operates and where new digital demands extend beyond it.

**Fig. 1:**
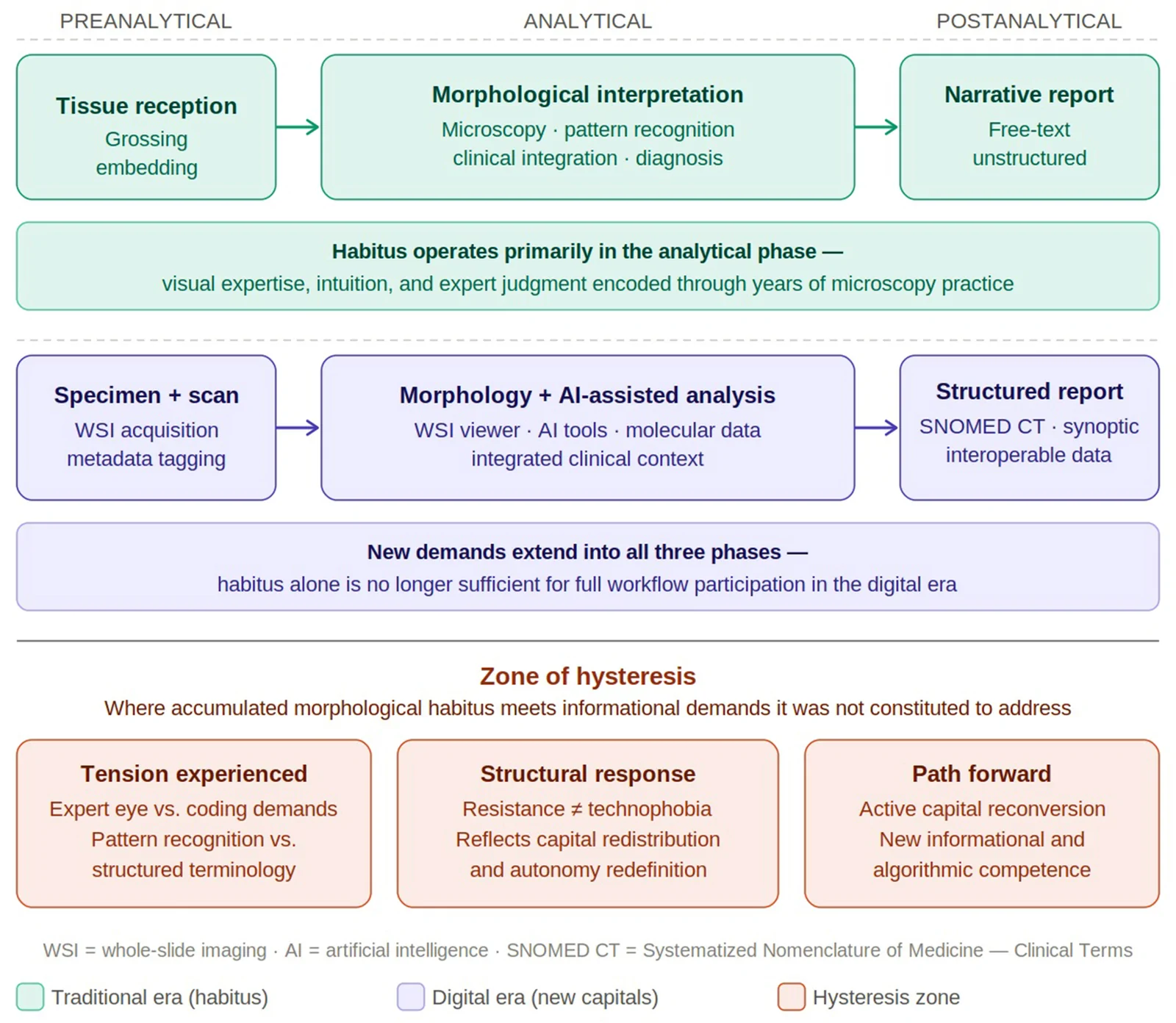
Habitus, workflow phases, and structural hysteresis in anatomic pathology. The figure contrasts the traditional era (teal) and the digital era (purple) across the three phases of the anatomic pathology workflow: preanalytical, analytical, and postanalytical. In the traditional era, the pathologist's habitus operates primarily in the analytical phase, where visual expertise, intuition, and expert judgment are embodied and applied. In the digital era, new informational demands extend across all three phases, producing a zone of structural mismatch (hysteresis, coral) in which accumulated morphological capital does not automatically transfer to the requirements of digital workflows. The zone of hysteresis is characterized by three coexisting states: tension experienced (expert eye vs coding demands), structural response (resistance as capital conflict), and path forward (active capital reconversion). AI: artificial intelligence; SNOMED CT: Systematized Nomenclature of Medicine — Clinical Terms; WSI: whole-slide imaging.

### Reconfiguration of symbolic authority

The transition from glass to pixel does not eliminate the pathologist's function. It redefines their position in the field.^8^ Professional excellence shifts from the exclusivity of the expert morphological eye toward the capacity to critically validate information in technologically mediated environments. Symbolic capital is reconfigured; it depends less on the singularity of individual judgment and more on the ability to navigate and curate complex informational ecosystems.

This restructuring implies the transformation, not the disappearance, of the habitus. The pathologist of the digital era is both a morphological interpreter and a producer and curator of structured data feeding clinical, epidemiological, and algorithmic knowledge systems.

### The pathology report as underestimated capital

The anatomopathological report has historically taken the form of an unstructured narrative text: a highly individualized integration of morphological observations, clinical context, and expert judgment. From a Bourdieusian perspective, this report is an objectification of the habitus — the form in which incorporated cultural capital is externalized as a communicable product. Its low structural formalization, however, has limited its value as reusable, machine-processable data.

The emergence of synoptic reporting — structured, checklist-based templates such as those developed by the College of American Pathologists (CAP) — represents the most concrete institutional expression of this transition. Synoptic reports impose standardized data fields, controlled vocabulary, and required elements. They constrain the expressive freedom of the narrative report while dramatically improving data completeness, reproducibility, and downstream utility. In Bourdieusian terms, they formalize the field's new rules: informational capital made mandatory.^15^

Digitalization reconfigures the equation further. The growing integration of health information systems, the need for interoperability, and the incorporation of AI^3^^,^^16^ transform data into a central resource — transitioning from an artisanal, individually integrated model toward a structured, reproducible, and formalized one. This constitutes the emergence of structured informational capital, a form whose value does not reside in the authority of the agent who produces it, but in its capacity to circulate and be processed within digital ecosystems.

### Data is not neutral: codification, ontologies, and power

Data does not pre-exist the process of knowledge production. It is the result of selection, classification, and codification — operations deeply embedded in power relations.^17^^,^^18^ Controlled terminology systems such as the Systematized Nomenclature of Medicine — Clinical Terms (SNOMED CT)^19^ and interoperability standards impose the need to translate diagnostic complexity into discrete, standardized, computable units. Standardization does not eliminate interpretation. It displaces it toward earlier stages of the process, where ontologies and classification systems are defined — frequently by actors external to clinical practice.

NLP and AI models^16^^,^^20^ further transform unstructured narrative reports into structured data, introducing additional layers of interpretation in which criteria of relevance are defined by models trained on prior corpora. The digital anatomopathological datum is the result of a double mediation: the pathologist's habitus and the coding systems that translate interpretation into structured format.

In Bourdieu's terms,^8^^,^^21^ the struggle for diagnostic legitimacy increasingly shifts toward the definition of what counts as valid data, how it must be encoded, and who holds the authority to interpret it. The assertion that “data are not neutral” carries a precise meaning here: data is a form of capital whose construction is traversed by epistemological, technological, and political decisions.

### Autoethnographic reflection: the pathologist at the threshold

Autoethnography positions the researcher's subjective experience as a legitimate source of sociological knowledge.^9^^,^^10^ The author of this work occupies a threshold position: more than fifteen years of practice as a pathologist in a laboratory of anatomic pathology based on conventional optical microscopy, combined with current training as a specialist in medical informatics. This dual belonging is an epistemologically privileged condition. It allows simultaneous observation from within and from the margin of the field.

The habitus described here is not a theoretical abstraction. It is a lived bodily experience: thousands of hours at the microscope, the reflexive adjustment of focus, the simultaneous reading of multiple tissue planes, and the immediate integration of morphological image and diagnostic hypothesis. This incorporated knowledge constitutes the core of professional identity and the primary source of authority in the field.

Simultaneous training in medical informatics introduces a disruptive lens. The daily diagnostic process becomes legible also as an unstructured information flow: observations, inferences, and decisions occurring in the pathologist's cognitive space without leaving a formalized record beyond the final narrative report. When observing a histological preparation, the question shifts from “what diagnosis corresponds?” to also include: “how is this finding coded? What SNOMED CT term represents it? What is lost in that translation?”

This experience concretely illustrates Bourdieu's hysteresis^4^: the visual habitus continues to function with full diagnostic effectiveness, but new field conditions — demand for interoperability, data structuring, integration with information systems — which introduce requirements the habitus alone was not constituted to address. The tension is not rejection of technology. It is the awareness that accumulated morphological capital does not automatically translate into competence within the new informational rule space of the digital field.

The threshold position is epistemologically productive. It makes visible what neither the purely technical pathologist nor the informatician without clinical training can see separately: that the digital transition in anatomic pathology is not a problem of technological adoption, but a transformation in the very conditions of production, legitimation, and circulation of diagnostic knowledge.

## Conclusion

The digitalization of anatomic pathology must be analyzed as a profound restructuring of the medical field — not merely as technological innovation. Nearly two centuries of methodological stability have configured a highly sedimented professional habitus whose symbolic authority was based on the bodily incorporation of knowledge and the intergenerational repetition of the act of looking. The current transition breaks that historical continuity and compels the redistribution of capitals within the field.

Data, historically a byproduct of the diagnostic act, emerges as a new form of capital. Its construction is not neutral. It is traversed by epistemological, technological, and political decisions. The pathologist is increasingly both a morphological interpreter and a producer and curator of structured data feeding clinical, epidemiological, and algorithmic knowledge systems.

The autoethnographic dimension of this work demonstrates that this transformation is not only structural but profoundly subjective — lived from within by those who simultaneously inhabit the morphological tradition and the demands of informational modernity.

The framework proposed here is theoretical and autoethnographic in nature.^9^^,^^10^ It invites empirical follow-up: survey-based studies of pathologist attitudes toward digital transition, qualitative research on change experiences across career stages, and institutional analyses of structured reporting adoption could all provide the evidential base that this essay, by design, does not claim to supply. The value of this work lies in naming and conceptualizing a structural phenomenon — hysteresis in anatomic pathology — that practitioners are already living but that the literature has not yet systematically theorized. The future of the pathologist will depend on the capacity to redefine symbolic capital at the intersection of subject, device, and algorithm.

## Declaration of generative AI and AI-assisted technologies in the writing process

During the preparation of this manuscript, the author used Claude (Anthropic) to assist with structural organization, language editing, and academic prose refinement, in accordance with Elsevier's generative AI policy for journals. All theoretical arguments, intellectual content, autoethnographic material, and conclusions are entirely the author's own. The author reviewed, edited, and takes full responsibility for all content as submitted.

## Funding

This research received no specific grant from any funding agency in the public, commercial, or not-for-profit sectors.

## Declaration of competing interest

The author declares that there are no competing interests, financial or non-financial, that could have influenced the design, development, or conclusions of this work.
